# Molecular regulation of chronic stress responses in European sea bass, *Dicentrarchus labrax*

**DOI:** 10.3389/fendo.2025.1611667

**Published:** 2025-06-26

**Authors:** Athanasios Samaras, Spyridon Kollias, Michail Pavlidis

**Affiliations:** ^1^ Fish Physiology Laboratory, Department of Biology, University of Crete, Heraklion, Greece; ^2^ Center for Ecological and Evolutionary Synthesis, Department of Biosciences, University of Oslo, Oslo, Norway

**Keywords:** chronic stress, cortisol, cortisone, HPI axis, stress response

## Abstract

**Introduction:**

This study examines the effects of predictable repeated chronic stress on the stress response and cortisol metabolism in European sea bass.

**Methods:**

Fish were exposed to daily stress for 11 days and sampled the next day before or after an additional stressor. Chronically stressed fish showed an attenuated acute cortisol response and altered circulating cortisone levels.

**Results and Discussion:**

Gene expression analyses revealed stress-induced regulatory changes. In the brain, *pomc* and *bdnf* mRNA expression was affected by chronic stress, while c*rf *by acute stress. In the head kidney, *gr2* was affected by both stress types, whereas *gr1* and *mr* responded only to acute stress. Neither *mc2r*, encoding the ACTH receptor, nor *hsd11b2*, responsible for cortisol inactivation, were affected. In the liver, *gr2* and *hsd11b2* were upregulated under chronic stress, suggesting an adaptive mechanism to regulate cortisol metabolism. In contrast, gill receptor expression remained largely unchanged, except for acute stress-induced *gr2*, *gr1*, and *mr* downregulation in chronically stressed fish, potentially modulating cortisol signaling. These findings suggest that chronic stress alters neuroendocrine regulation, desensitizing the HPI axis and impairing the acute stress response. Understanding these mechanisms provides insights into chronic stress adaptation in fish, with implications for aquaculture and stress physiology research.

## 1 Introduction

Chronic stress has been widely recognized for its detrimental effects on animal health and behavior (1, 2), and fish are no exception (3). Chronic stress can be described as a prolonged or repeated exposure to stressors that exceed an organism’s capacity to cope through allostatic processes, leading to physiological dysregulation and increased allostatic load (2). Unlike acute stress, which is a short-term reaction to immediate threats, chronic stress persists over an extended period, potentially leading to maladaptive outcomes such as impaired growth (4, 5), as well as immune function, and overall health (3, 6). Predictable, repeated chronic stress is crucial in shaping physiological and behavioral adaptations in animals, including fish, as it allows for the development of coping mechanisms that mitigate adverse effects (7). While much of the research on stress responses has focused on terrestrial species, the impact of chronic stress on aquatic organisms, particularly fish, has become a growing area of interest in aquaculture as well as environmental and behavioral fish biology. Fish, especially under an intensive farming environment, are frequently exposed to a variety of stressors, including environmental ones, such as changes in water quality and temperature fluctuations, but mainly production-related activities such as husbandry practices and high stocking density exposure. These stressors can trigger a cascade of physiological responses, which, when persistent, may lead to habituation or dysregulation of the physiological mechanisms of the fish (3, 8, 9).

The neuroendocrine system, particularly the hypothalamic-pituitary-interrenal (HPI) axis, plays a central role in mediating the stress response in fish, influencing a range of physiological processes such as immune function, metabolism and growth, and osmoregulation (9, 10). Despite advances in understanding the acute stress response in fish (9, 11, 12), the long-term effects of chronic stress remain underexplored. Prolonged activation of the HPI axis due to chronic stress affects physiological processes in species-specific ways, influenced also by the nature and duration of the stress protocol. While chronic stress often leaves circulating cortisol levels unchanged (4, 5, 13–18), both attenuated (19–21) and elevated levels (22–25) have been reported. Additionally, chronic stress can impair a fish’s ability to cope with future stressors; as evidenced by a blunted cortisol response following acute stress in previously stressed individuals (4, 5, 18, 26).

Beyond the effects of chronic stress on the final output of the HPI axis, understanding the regulatory mechanisms governing this axis is also critical. However, to date, only a limited number of studies have investigated this aspect. The HPI regulation begins in the pre-optic area of the hypothalamus, where *crh* is expressed and CRH is produced and secreted to stimulate the expression of *pomc* and subsequent production of ACTH from the pituitary (9). Subsequently, ACTH binds to the MC2R receptor in the head kidney to signal the production and secretion of cortisol. The studies performed so far in Atlantic salmon (*Salmo salar*) exposed to predictable (27) or unpredictable (4, 20) chronic stress have shown increased *crh* expression. In a similar manner, exposure to a predictable stress protocol was shown to cause an increase in CRH levels depending on the duration of the chronic stress (25). Moreover, in the same species predictable chronic stress was shown to cause an increase in circulating ACTH levels (25), while unpredictable stress was shown to cause either an increase on the expression of *pomca1* and *pomcb* in the pituitary of fish at the parr stage (4), or to have no effect on juveniles (20). In rainbow trout (*Oncorhynchus mykiss*), it has been shown that social chronic stress leads to chronic elevation of cortisol levels in subordinate fish. The elevated cortisol levels result from increased cortisol production by the head kidney under baseline conditions; however, this response is attenuated when the tissue is stimulated during *in vitro* testing (28). Finally, in European sea bass (E. sea bass; *Dicentrarchus labrax*) exposure to different intensity of chronic stress has caused an increase of *crh* expression from the pre-optic area only at the highest intensity, while *pomc* expression in the pituitary was reduced in all chronic stress groups compared to control fish (5). On the other hand, gilthead seabream (*Sparus aurata*) exposed to the same chronic stress protocol as E. sea bass, displayed no change in the expression of either *crh* or *pomc*. Together, these findings underscore the interspecific and protocol-dependent differences in HPI axis regulation in fish under chronic stress. In the mammalian brain, the brain-derived neurotrophic factor (BDNF) plays a key role in the stress response by interacting with the HPA axis and glucocorticoid signaling, influencing brain structure through processes like neurogenesis, synaptic plasticity, and dendritic spine formation (29, 30). In fish, *bdnf* expression has been reported to be upregulated in acutely stress gilthead seabream larvae (31). Regarding chronic stress, it has been reported to cause increased (26) or unaltered (32) *bdnf* expression in zebrafish (*Danio rerio*) brain.

While much attention has been given to the regulation at the brain level, few studies have investigated the effects on interrenal tissue or on peripheral tissues such as the liver and gills. Specifically, the *11hsd2b* gene encodes an enzyme responsible for converting active cortisol into its inactive form, cortisone. Madaro et al. (4) have shown no differential expression of this gene in Atlantic salmon (*Salmo salar*) exposed to chronic stress. On the contrary, in common carp (*Cyprinus carpio*), acute net stress has been associated with elevated expression of *11hsd2b* in the same tissue (33). Additionally, this gene exhibits differential expression in E. sea bass, with lower expression in individuals characterized by low cortisol responsiveness compared to highly responsive fish from the same population (34). Moreover, in Atlantic salmon the mRNA levels of the ACTH receptor gene (*mc2r*) as well as genes involved in cortisol synthesis (*star*) or signaling (*gr1, gr2, mr*) were also unaffected by chronic stress (4). However, in rainbow trout, daily stress exposure has been associated with increased expression of both glucocorticoid receptors, *gr1* and *gr2* (35). The ratio of the glucocorticoid to the melanocorticoid receptors (*gr1/mr* and *gr2/mr*) is also of interest, as it has been shown to be affected by chronic stress in mammals (36) and fish (5, 37). This study characterized the gene expression profile underlying chronic stress responses in HPI-related, and peripheral tissues of E. sea bass (*Dicentrarchus labrax*). Fish were subjected to a predictable chronic stress protocol, and cortisol and cortisone levels, along with the expression of stress-related regulatory genes, were analyzed before and after an additional acute stressor, in comparison to unstressed control fish.

## 2 Materials and methods

### 2.1 Animals and experimental design

In the present study a predictable repeated stress protocol was developed and applied in E. sea bass fish as previously described in Samaras et al. (18), as the results presented here are part of the same experiment. In brief, 28 E. sea bass of average weight 191.4 ± 36.4 g were divided in 4 × 250 l (7 fish per tank, stocking density of 5.36 kg m^3^) to maintain appropriate stocking density. Out of these, 6 fish in total were randomly sampled, as described below. The system used open flow tanks with a renewal rate of approximately 75% per hour. The experiment was performed at the Hellenic Centre for Marine Research (HCMR) and during the whole period temperature was kept at 19°C, the photoperiod was set at 12L:12D and salinity was 37.

Following a two-week acclimation phase, a chronic stress protocol was introduced. Two tanks of fish were designated as the control group and remained unstressed throughout the experiment (“Chronic +” group). In contrast, fish in two other tanks were subjected to the experimental stress conditions outlined below (“Chronic -” group). Given that E. sea bass tend to significantly reduce or completely stop feeding under stress, neither the control nor the stressed fish were fed during the study to eliminate potential dietary influences. According to Samaras et al. (18), no significant differences were found in most stress-related physiological biomarkers between fed and unfed control groups, apart from plasma glucose levels which were significantly higher in the fed group.

The chronic stress protocol consisted of subjecting the fish to a daily stressor between 11:00 and 12:00, using one of two acute stress methods: (i) chasing with a net for 5 minutes or (ii) confinement in one-fifth of the tank for 30 minutes. The protocol alternated between the two stressors to better mimic the variability of stressors that fish may encounter in an aquaculture environment and also to avoid habituation to a single type of stress. To ensure consistency in stress intensity, the same person carried out the chasing procedure, while confinement was achieved by reducing the water volume to a predetermined level, marking one-fifth of the original tank volume. These stressors were alternated each day over an 11-days period. On the 12th day, fish from both the control and chronically stressed groups were sampled immediately (“no acute” fish; n = 3 per replicate/6 per group) and euthanized using a high dose of anesthesia (phenoxyethanol, 500 ppm). The remaining fish were subjected to an additional stressor—5 minutes of chasing—and sampled one hour later (“Acute +” fish; n = 3 per replicate/6 per group), when cortisol levels typically peak in this species (11). Blood was drawn from the caudal vessel using heparinized syringes, and plasma was separated by centrifugation at 2,000g for 10 minutes before being stored at -20°C for later analysis. Fish were then immediately dissected for the collection of whole brain, including the pituitary, head kidney, liver, and gill samples, which were snap frozen in liquid nitrogen and stored at -80°C until the analysis.

### 2.2 Analytical procedures

Plasma cortisol and cortisone levels were measured using commercially available ELISA kits (DRG^®^ Cortisol ELISA, DRG^®^ International Inc., Germany; Abbexa Cortisone ELISA, Abbexa, UK). The performance of the cortisol kit has been previously validated for E. sea bass (38), and the plasma cortisone kit was evaluated with linearity tests using sequential dilutions of pooled fish plasma samples (r = 0.945; *n* = 3).

### 2.3 RNA purification and cDNA synthesis

Tissue samples (20–30 mg) were disrupted and homogenized using the TissueRuptor (Qiagen, Hilden, Germany) for 20 s in 350 μl LBP lysis buffer (NucleoSpin^®^ RNA Plus, MACHEREY-NAGEL GmbH & Co. KG, Duren, Germany). Total RNA was isolated using the NucleoSpin^®^ RNA Plus kit (MACHEREY-NAGEL GmbH & Co. KG, Duren, Germany), according to manufacturers’ instructions. RNA yield and purity were determined by measuring the absorbance at 260 and 280 nm, using Nanodrop^®^ ND-1000 UV-Vis spectrophotometer (Peqlab, Erlangen, Germany), while its integrity was tested by 1% agarose gel electrophoresis. Reverse transcription (RT) was performed using 1 μg RNA with the PrimeScript Reverse Transcription Reagent Kit with gDNA Eraser (Takara Bio, USA) following the manufacturer’s instructions. Due to insufficient RNA yield and purity one brain and head kidney sample, as well as two liver samples were not processed.

### 2.4 Quantitative real-time PCR

The mRNA expression of genes encoding for corticotropin releasing factor (*crf*), proopiomelanocortin (*pomc*), brain-derived neurotrophic factor (*bdnf*), glucocorticoid receptors (*gr1*, *gr2*), mineralocorticoid receptor (*mr*), melanocortin receptor 2 (*mc2r*), and 11-β-dehydrogenase type 2 (*hsd11b2*), was determined with real time quantitative polymerase chain reaction (qPCR) assays using the LightCycler^®^ 480 SYBR Green I Master (Roche, Norway). The oligonucleotides used in the qPCR analysis are shown in **Table 1**. The qPCR reactions were performed in a LightCycler^®^ 480 thermocycler (Roche, Norway) in 384 well plate format, under the following parameters: (1) 95°C for 3 min (HotStarTaq DNA Polymerase activation step), (2) 94°C for 15 s (denaturation step), (3) 60°C for 30 s (annealing step), (4) 72°C for 20 s (extension step), cycling steps (2) to (4) for 40 cycles. All samples were run in triplicate, and minus reverse transcriptase (-RT), no template and positive controls were included in each plate. A relative standard curve was constructed for each gene, using 4 sequential dilutions (1:5) of a pool of all the cDNA samples. To normalize gene expression, three references genes: actin beta 1 *(actb1)*, eukaryotic translation elongation factor 1A *(eef1a)*, and *18s*, were initially accessed for the stability of their expression under the current experimental conditions using *GeNorm* (42). The two more stable reference genes based on the geNORM analysis were *actb1* and *eef1a* and were therefore used for the normalization of target genes.

**Table 1:** Primer sequences used in qPCR in the current study.

Gene	Forward Primer 5’ to 3’	Reverse Primer 5’ to 3’	Reference	Acc. no	Efficiency
*actb1*	CGCGACCTCACAGACTACCT	AACCTCTCATTGCCGATG	(39)	AJ493428	2.09
*eef1a*	GCCAGATCAACGCAGGTTACG	GAAGCGACCGAGGGGAGG	(39)	FM019753	1.98
*18s*	TCAAGAACGAAAGTCGGAGG	GGACATCTAAGGGCATCACA	(40)	AM419038	2.00
*crf*	ATCACCTGCCATCTACAA	TGATGTTCCCAACTTTCC	(39)	JF274994	1.89
*pomc*	CCGGTCAAAGTCTTCACCTC	ACCTCCTGTGCCTTCTCCTC	(41)	*	1.88
*gr1*	GAGATTTGGCAAGACCTTGACC	ACCACACCAGGCGTACTGA	(40)	AY549305	1.99
*gr2*	GACGCAGACCTCCACTACATTC	GCCGTTCATACTCTAACCAC	(40)	AY619996	2.03
*mr*	CCTGTCTCCTCATGAATGG	AATCTGGTAATGGAATGAATGTC	(39)	JF824641	2.04
*hsd11b2*	CACCCAGCCACAGCAGGT	ACCAAGCCCCACAGACC	(39)	*	2.01
*mc2r*	CATCTACGCCTTCCGCATTG	ATGAGCACCGCCTCCATT	(41)	FR870225	1.90
*bdnf*	CATCGGACGGAAGGCTACT	GCTCCTCTATCACCTGCTCAA	(41)	FJ711591.1	2.05

*Primers for these genes were designed based on the conserved regions as revealed by multiple sequence alignments of other teleost fish. The products of each primer pair were further checked with sequencing to confirm amplification of the desired genes.

### 2.5 Statistical analysis

Statistical analysis and graphical analysis were performed using GraphPad Prism 8 (GraphPad Software) and RStudio. All data are presented as means ± standard deviation (S.D.). Data were initially checked for normality and homogeneity of variance using the Kolmogorov-Smirnov and Levene tests, respectively. Outliers were identified as extreme values using Grubbs’ test, using strict criteria (α = 0.01) to ensure control given the small sample size. In total, two outliers were identified in brain *pomc* expression and one in brain *bdnf* and head kidney *gr1*, all being extremely higher than the rest of the samples. Two-way Analysis of Variance (ANOVA) was performed using *chronic stress* and *acute stress* as factors, also testing their interaction. The Principal Component Analysis (PCA) was performed for the gene expression of the HPI-related tissues, brain and head kidney, in RStudio using the *factoextra* and *FactoMineR* packages. Tissues were analyzed separately in order to identify tissue-specific effects of chronic and acute stress. The ratios between cortisol receptors, *gr1*, *gr2* and *mr* were not used in this analysis due to strong correlation between them and the expression of the receptors themselves. All data were normalized prior to analysis.

### 2.6 Ethical approval

All procedures have been approved by the Departmental Animal Care Committee (protocol code 130/2020) following the Three Rs principle, in accordance with Greek (PD 56/2013) and EU (Directive 63/2010) legislation on the care and use of experimental animals. All experimental procedures were performed by FELASA accredited researchers.

## 3 Results

### 3.1 Stress hormones

**Figure 1:**
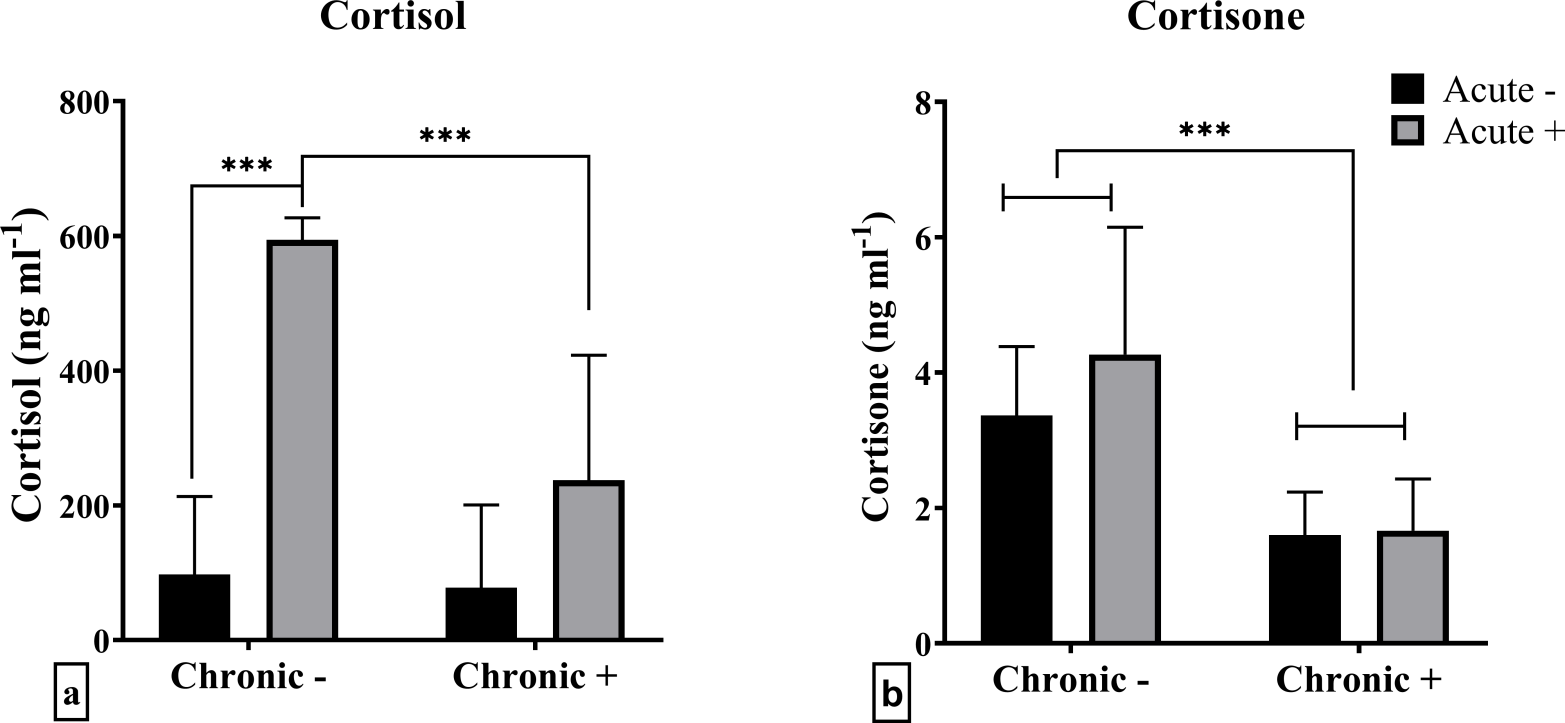
Mean ± SD plasma concentrations of **(a)** cortisol and **(b)** cortisone in European sea bass under chronic (“Chronic +”) and acute (“Acute +”) stress or control (“Chronic -” and “Acute -”) conditions. Asterisks indicate significant differences between groups, as shown by connecting lines (*p < 0.05, **p < 0.01, ***p < 0.001). *n* = 6/group.

The plasma cortisol results originate from the same experiment previously reported by Samaras et al. (18). They are shown again here using a reduced dataset that includes only the fish (n = 6 per treatment) that were dissected for gene expression analysis, to ensure consistency across measured parameters. A significant interaction between the factors chronic and acute stress was observed in cortisol levels (F_1,20_ = 10.69; *p* = 0.004) (**Figure 1a**). There were no significant differences in mean cortisol concentrations between control (Chronic -) and chronically stressed fish, that were not subjected to an extra acute stressor. Additionally, while post-acute stress cortisol levels were elevated in non-chronically stressed fish, this effect was absent in chronically stressed fish. Cortisone levels, on the other hand, were only affected by chronic stress (F_1,20_ = 20.56; *p* < 0.001), specifically being lower in chronically stressed fish (**Figure 1b**).

### 3.2 mRNA abundance

**Figure 2:**
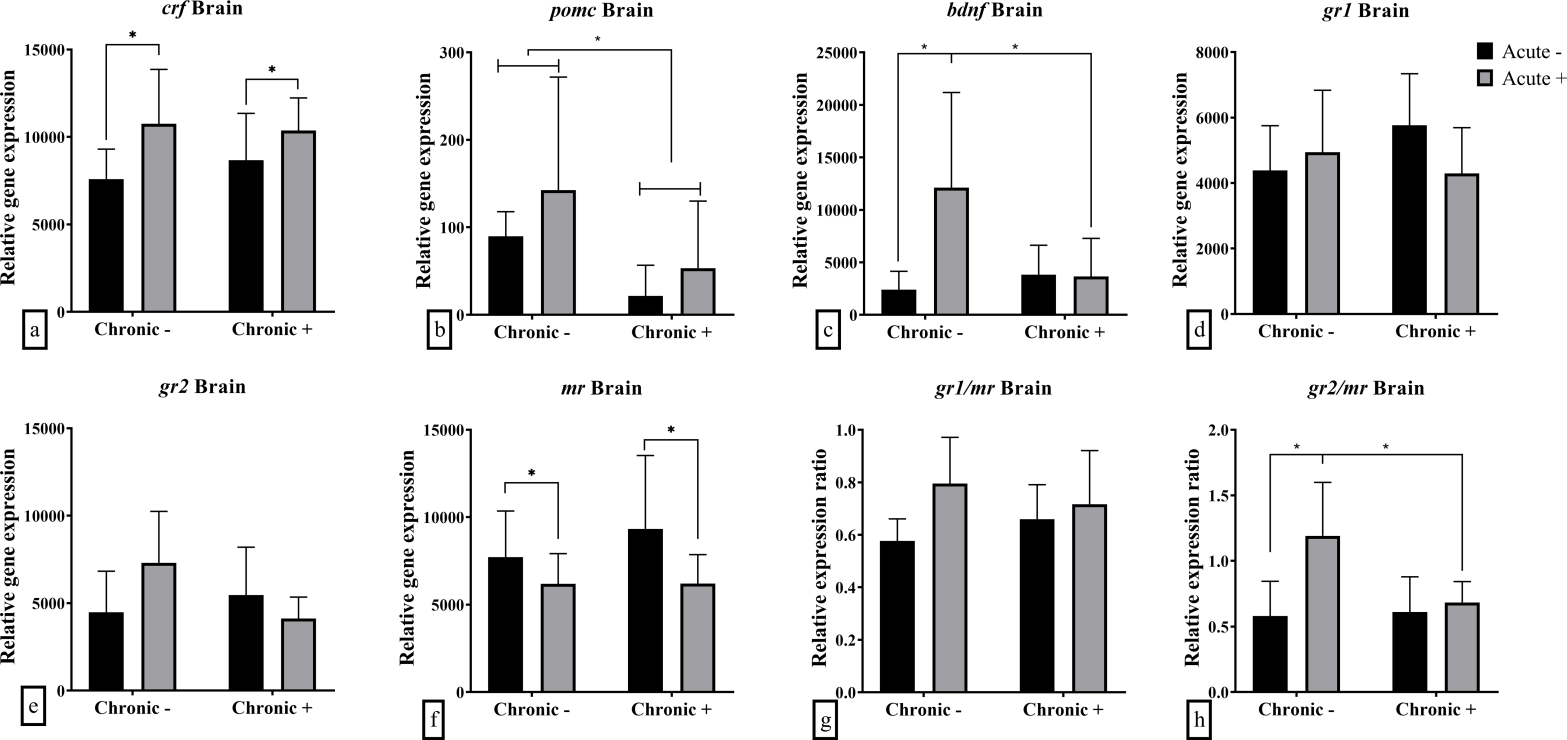
Mean ± SD whole brain relative mRNA abundance for **(a)** *crf*, **(b)** *pomc*, **(c)** *bdnf*, **(d)** *gr1*, **(e)** *gr2*, **(f)** *mr* and the ratio of **(g)** *gr1/mr* and **(h)** *gr2/mr* in European sea bass under chronic (“Chronic +”) and acute (“Acute +”) stress or control (“Chronic -” and “Acute -”) conditions. Asterisks indicate significant differences between groups, as shown by connecting lines (*p < 0.05, **p < 0.01, ***p < 0.001). n = 6/group, apart from the “Chronic +/Acute -” group where n = 5 in all genes except for *pomc* were n = 4. Additionally, n = 5 for “Chronic -/Acute +” in *pomc* and *bdnf*.

mRNA abundance analysis of whole brain samples revealed that chronic stress, acute stress, and their interaction influenced the mRNA abundance of certain genes. Specifically, the effect of chronic stress was highlighted in the down-regulated mRNA abundance of brain *pomc* (F_1,17_ = 5.164; *p* = 0.036) (**Figure 2b**), while acute stress resulted in up-regulated *crf* (F_1,19_ = 5.875; *p* = 0.026), and down-regulated *mr* (F_1,19_ = 4.384; *p* = 0.049) mRNA abundance (**Figures 2a, f**). The mRNA abundance of *bdnf* (F_1,18_ = 5.855; *p* = 0.027) and the ratio of *gr2/mr* (F_1,19_ = 4.929; *p* = 0.038) were affected by the interaction of chronic and acute stress (**Figures 2c, h**). Specifically, it was shown that chronic stress led to decreased mRNA abundance of *bdnf* and the *gr2/mr* ratio in acutely stressed fish compared to non-chronically stressed fish. *gr1, gr2* mRNA abundance and *gr1/mr* ratio were not affected by either acute or chronic stress (**Figures 2d, e, g**).

**Figure 3:**
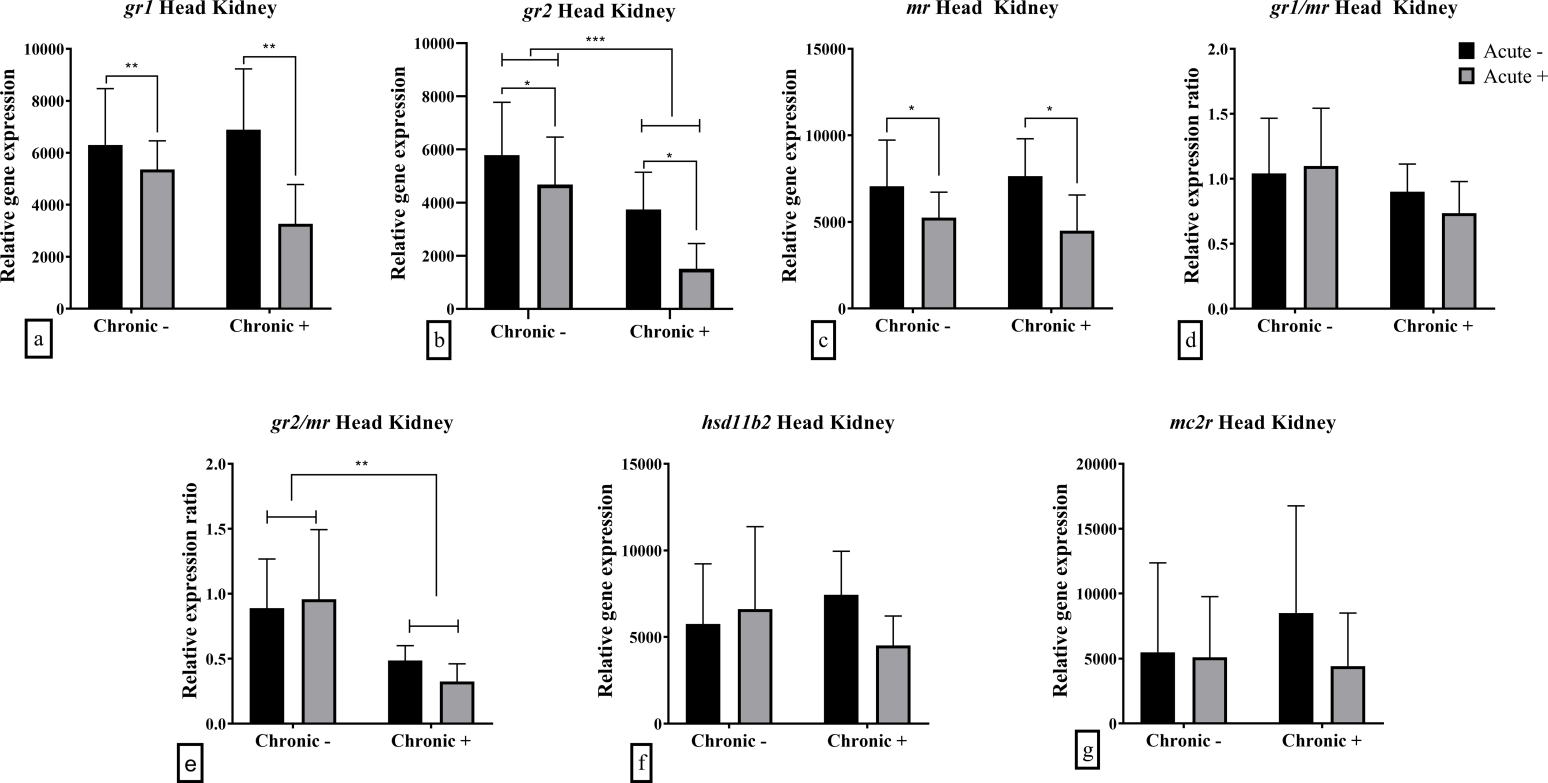
Mean ± SD head kidney relative mRNA abundance for **(a)** *gr1*, **(b)** *gr2*, **(c)** *mr*, **(d)** *gr1/mr* ratio, **(e)** *gr2/mr* ratio, **(f)** *hsd11b2* and **(g)** *mc2r* in European sea bass under chronic (“Chronic +”) and acute (“Acute +”) stress or control (“Chronic -” and “Acute -”) conditions. Asterisks indicate significant differences between groups, as shown by connecting lines (*p < 0.05, **p < 0.01, ***p < 0.001). n = 6/group, apart from the “Chronic +/Acute -” group where n = 5 in all genes and “Chronic -/Acute -” group for *gr1* and the associated *gr1/mr*.

In the head kidney, chronic stress caused a down-regulation in the mRNA abundance of *gr2* (F_1,19_ = 15.30; *p* < 0.001) and *gr2/mr* ratio (F_1,19_ = 12.61; *p* = 0.002) (**Figures 3b, e**). Acute stress also had a down-regulating effect in *gr1* (F_1,18_ = 8.836; *p* = 0.008), *gr2* (F_1,19_ = 6.273; *p* = 0.022) and *mr* (F_1,19_ 7.739; *p* = 0.012) mRNA abundance (**Figures 3a-c**). Neither *hsd11b2* nor *mc2r* were affected by acute or chronic stress or their combination (**Figures 3f, g**). *gr1/mr* ratio, as well as *hsd11b2* and *mc2r* mRNA abundance were not influenced by acute and chronic stress (**Figures 3d, f, g**).

**Figure 4:**
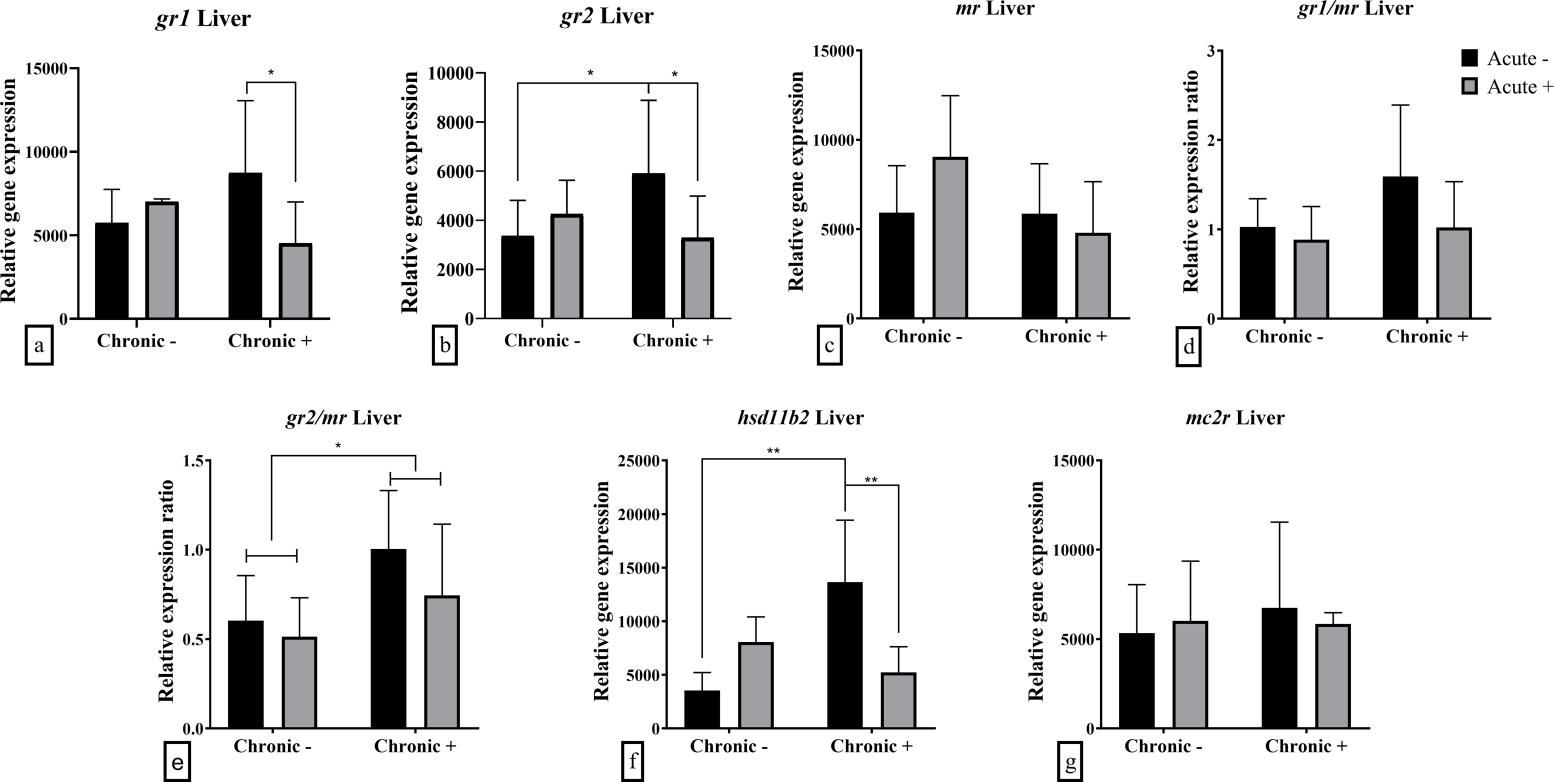
Mean ± SD hepatic relative mRNA abundance for **(a)** *gr1*, **(b)** *gr2*, **(c)** *mr*, **(d)** *gr1/mr* ratio, **(e)** *gr2/mr* ratio, **(f)** *hsd11b2* and **(g)** *mc2r* in European sea bass under chronic (“Chronic +”) and acute (“Acute +”) stress or control (“Chronic -” and “Acute -”) conditions. Asterisks indicate significant differences between groups, as shown by connecting lines (*p < 0.05, **p < 0.01, ***p < 0.001). n = 6/group, apart from the “Chronic +/Acute -” group and the “Chronic +/Acute +” where n = 5 in all genes.

The hepatic mRNA abundance of glucocorticoid receptors was influenced by the interaction between acute and chronic stress. Specifically, both *gr1* (F_1,18_ = 6.191; *p* = 0.023) and *gr2* (F_1,18_ = 4.527; *p* = 0.047) were down-regulated following acute stress in chronically stressed fish (**Figures 4a, b**). Additionally, *gr2* mRNA abundance was up-regulated in chronically stressed fish prior to acute stress compared to non-chronically stressed fish. The *gr2/mr* ratio was significantly affected by chronic stress (F_1,18_ = 6.056; *p* = 0.024), with higher values observed under chronic stress (**Figure 4e**). Similarly, hepatic *hsd11b2* mRNA abundance was influenced by the interaction of acute and chronic stress (F_1,18_ = 13.910; *p* = 0.002), showing up-regulation in chronically stressed fish that were not exposed to acute stress compared to both non-stressed and dually stressed fish (**Figure 4f**). The mRNA abundance of *mr* and *mc2r* as well as the ratio of *gr1/mr* were not affected by either acute or chronic stress exposure (**Figures 4c, d, g**).

**Figure 5:**
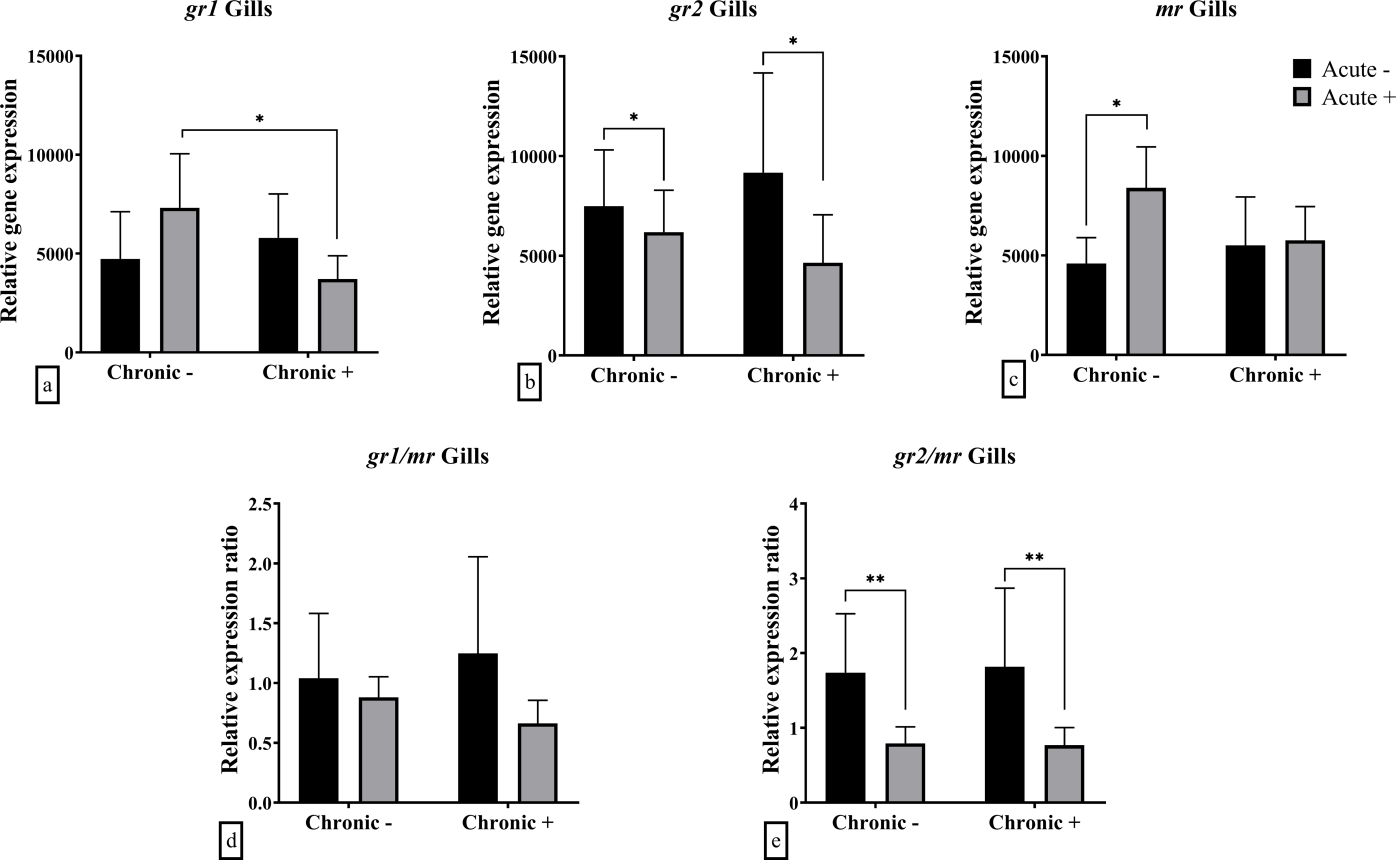
Mean ± SD gill gene expression for **(a)** *gr1*, **(b)** *gr2*, **(c)** *mr*, **(d)** *gr1/mr* ratio, **(e)** *gr2/mr* ratio in European sea bass under chronic (“Chronic +”) and acute (“Acute +”) stress or control (“Chronic -” and “Acute -”) conditions. Asterisks indicate significant differences between groups, as shown by connecting lines (*p < 0.05, **p < 0.01, ***p < 0.001). n = 6/group.

In the gills an interaction effect was observed in the mRNA abundance of *gr1* (F_1,20_ = 6.713; *p* = 0.018) and *mr* (F_1,20_ = 5.090; *p* = 0.036) (**Figures 5a, c**). *Post-hoc* analysis showed *gr1* mRNA abundance was downregulated in fish exposed to both acute and chronic stress compared to those exposed to acute stress alone. On the other hand, the *mr* mRNA abundance was up-regulated after exposure to acute stress in no-chronically stressed fish only. Finally, the mRNA abundance of *gr2* (F_1,20_ = 4.691; *p* = 0.043) and the ratio between *gr2/mr* (F_1,20_ = 13.01; *p* = 0.002) were lower in acutely stressed fish, in both non-chronically and chronically stressed fish (**Figures 5b, e**). The ratio *gr1/mr* ratio was not affected by the application of acute or chronic stress (**Figure 5d**).

### 3.3 PCA analysis

**Figure 6:**
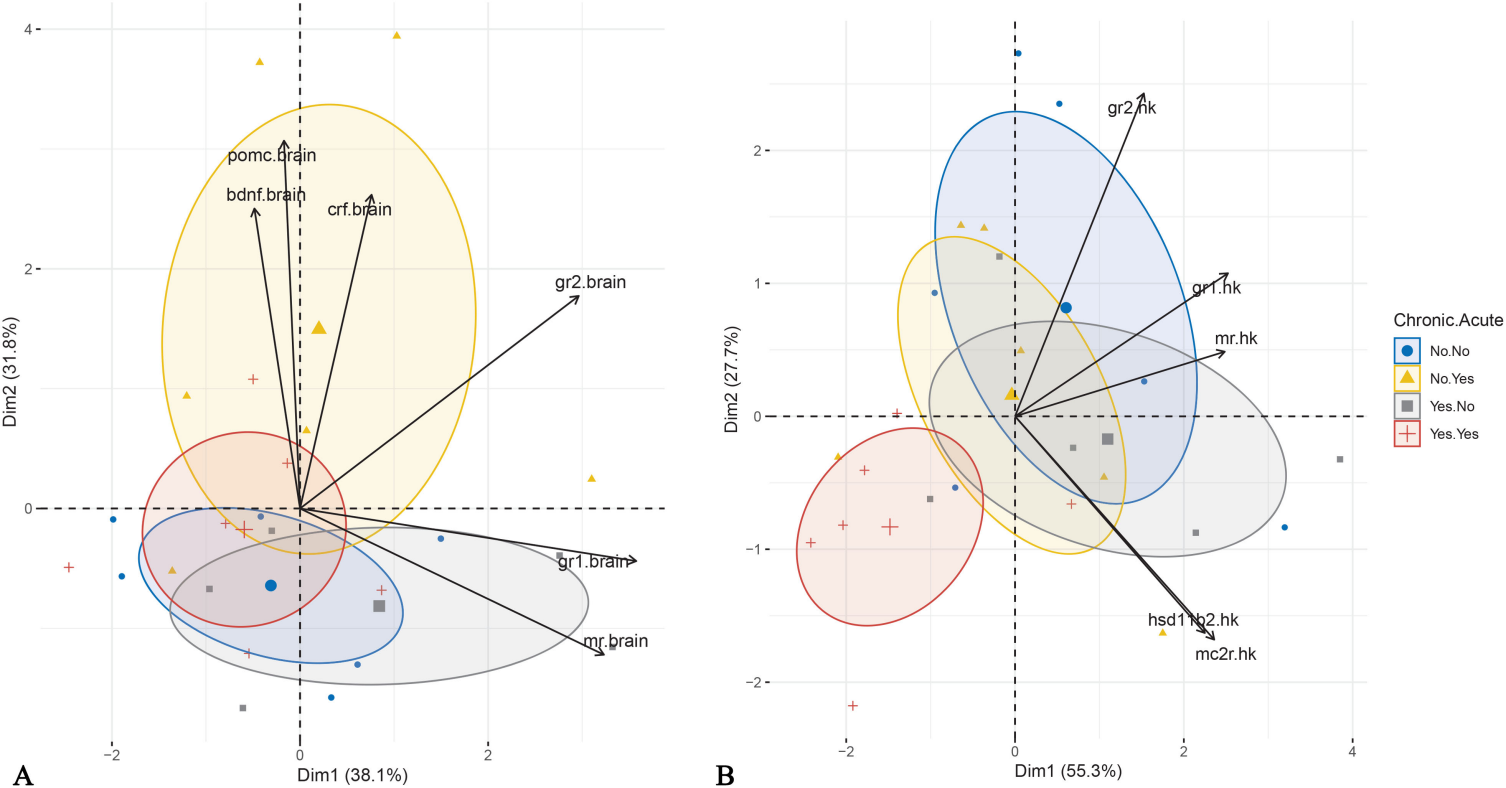
Principal Component Analysis performed for brain **(A)** and head kidney **(B)** gene expression data. The individuals are plotted based on the application of acute and chronic stress. Ellipse represents confidence ellipse.

PCA of brain mRNA abundance revealed that the first two principal components accounted for 69.9% of the total variance. The first component (PC1) had an eigenvalue of 2.288, explaining 38.1% of the variance, and was significantly correlated with *gr1*, *gr2*, and *mr* mRNA abundance (**Table 2**). The second component (PC2) had an eigenvalue of 1.906, explaining 31.8% of the variance, and was significantly associated with *pomc*, *bdnf*, *crf*, and *gr2* mRNA abundance (**Table 2**). Visualization of sample distribution along these components indicated that fish exposed to acute but not chronic stress were separated from other groups along PC2, driven primarily by *crf*, *pomc* and *bdnf* mRNA abundance (**Figure 6A**). In contrast, fish subjected to both chronic and acute stress were not clearly separated from those experiencing only chronic stress, though their centroid distribution exhibited a trend influenced primarily by *mr* and *gr1* mRNA abundance.

**Table 2:** Eigenvalues, % variance explained and correlation of variables to the first two principal components of the PCA for the brain and head kidney gene expression analysis.

Brain			Head Kidney		
Tissue	Comp 1	Comp 2	Tissue	Comp 1	Comp 2
Eigenvalue	2.288	1.906	Eigenvalue	2.764	1.384
% of variance	38.1	31.8	% of variance	55.3	27.7
*crf*	0.200	0.691*	*hsd11b2*	0.740*	-0.537*
*pomc*	-0.046	0.810*	*mc2r*	0.777*	-0.553*
*bdnf*	-0.128	0.661*	*gr1*	0.831*	0.354
*gr1*	0.944*	-0.116	*gr2*	0.502*	0.800*
*gr2*	0.782*	0.469*	*mr*	0.818*	0.160
*mr*	0.853*	-0.322			

An asterisk (*) indicates significant correlation between the respective variable and the Component.

In the head kidney, PCA showed that the first two components explained 83.0% of the total variance, with PC1 accounting for twice the variance of PC2 (**Table 2**). PC1 was positively influenced by *gr1*, *mr*, *mc2r*, *hsd11b2*, and *gr2*, while PC2 was driven positively by *gr2* and negatively by *hsd11b2* and *mc2r*. Visualization in Cartesian space demonstrated that fish exposed to both acute and chronic stress formed a distinct cluster, primarily driven by *gr2* but also *gr1* and *mr* mRNA abundance (**Figure 6B**).

## 4 Discussion

The present study demonstrated the effects of exposure to a predictable, repeated chronic stress protocol on the regulation of cortisol signaling and metabolism in various tissues of E. sea bass. As previously shown (5, 18) cortisol levels in chronically stressed E. sea bass were attenuated when fish were exposed to an additional acute stress challenge, compared to the non-chronically stressed fish. Interestingly, the present study showed that cortisone levels were exclusively influenced by chronic stress, showing a significant decrease in chronically stressed fish. Previous studies in coho salmon (*Oncorhynchus kisutch*) (43) and rainbow trout (44) have shown that acute stress increases cortisone levels in a time-dependent manner. Therefore, the lack of cortisone elevation observed in the present study may be attributed to the timing of sample collection relative to the stress event. This finding further underscores the distinct physiological effects of chronic stress, which may interfere with the normal hormonal regulation of stress responses.

These results suggest that chronic stress may lead to adaptive or maladaptive changes in the fish’s physiological stress response. The attenuation of the acute cortisol response in chronically stressed fish could reflect a form of desensitization or blunted reactivity of the HPI axis, which is a common physiological adaptation in response to prolonged stress (4, 5, 18, 26). There are two possible explanations for this attenuated response. The first is that chronically stressed fish may exhibit reduced sensitivity as a protective mechanism to avoid long lasting energy expenditure. Alternatively, the lack of an acute cortisol response in these fish may suggest that prolonged stress has impaired the normal negative feedback mechanisms of the hypothalamic-pituitary-interrenal (HPI) axis, possibly due to exhaustion. In contrast, fish not exposed to chronic stress appear to maintain a functional HPI axis, allowing for a typical cortisol response to acute challenges.

The present study investigated the mechanisms underlying the effects of repeated predictable chronic stress in E. sea bass in an attempt to decipher the mechanism responsible for the observed attenuated cortisol response. This was done through the analysis of the expression of key genes involved in HPI-axis regulation, cortisol signaling, and metabolism. mRNA abundance patterns in the brain and head kidney provided molecular insights into the physiological and hormonal adaptations to both chronic and acute stress. The PCA analysis of brain gene expression confirmed that acute stress induced transient changes in HPI-axis regulation only in non-chronically stressed fish. Fish exposed to acute stress without prior chronic stress exposure separated from the other groups along PC2, which was primarily influenced by *crf, pomc, and bdnf* mRNA abundance. This suggests that chronic stress modifies the brain’s acute stress response, potentially leading to significant alterations in downstream physiological outcomes, such as the absence of cortisol elevation. In contrast, differences in head kidney mRNA abundance show that combined chronic and acute stress affects mRNA abundance, likely through altered corticosteroid signaling. Fish exposed to both types of stress were clustered separately mainly along the PC1 due to reduced mRNA abundance of *gr2*, and to a lesser extent, *gr1* and *mr*. These findings support the hypothesis that chronic stress desensitizes the HPI axis at the brain level, diminishing the fish’s ability to mount an acute stress response.

In more details, in the brain the lower *pomc* mRNA abundance under chronic stress suggests reduced ACTH production, leading to attenuated cortisol signaling to the head kidney. In contrast, the up-regulated *crf* mRNA abundance following acute stress indicates typical HPI-axis activation in response to short-term stressors. However, this did not translate into a strong cortisol surge in chronically stressed fish, likely due to downstream suppression of *pomc*, potentially caused by receptor dysregulation, as shown by the reduced *gr2/mr* in acutely stressed fish previously exposed to chronic stress. In teleosts, two glucocorticoid receptors, GR1 and GR2, and one mineralocorticoid receptor, MR, mediate cortisol signaling. These receptors differ in their affinity to cortisol, with GR2, along with the mineralocorticoid receptor (MR), having a higher affinity for cortisol and is likely activated at low basal concentrations, while GR1 may be more responsive to elevated cortisol levels during acute stress (45–47). This differential sensitivity suggests distinct functional roles in stress regulation, which can help interpret tissue- and receptor-specific expression patterns observed in this study. In this context, studying their ratio can indicate differential regulation of important functions, and their disequilibrium has been associated with impaired appraisal, poor learning and fear avoidance in vertebrates (36, 48).

A similar response was noticed in the mRNA abundance of *bdnf*. Specifically, acute stress induced *bdnf* overexpression in non-chronically stressed fish, consistent with findings in gilthead seabream larvae (31). This increase, however, was absent in chronically stressed fish, as previously observed in E. sea bass following exposure to a predictable stressor (7).

A previous study on E. sea bass (5) found that low- to medium-intensity chronic stress did not affect *crf* mRNA abundance in the pre-optic area of the telencephalon, consistent with the present study. However, high-intensity stress increased *crf* mRNA abundance compared to controls. Moreover, regardless of stress intensity, *pomc* mRNA abundance was reduced, being in agreement with the current results. It should be noted that the protocols used in these studies differed, with the current protocol involving daily stressing of the fish, while the one presented at Samaras et al. (5) consisting of stressing the fish thrice per week. Although responses to chronic stress in vertebrate animals are influenced by factors such as intensity, duration, repeatability, but also predictability, and controllability (7, 49, 50), the consistency of results suggests a basic underlying chronic stress regulation mechanism in E. sea bass.

In Atlantic salmon, chronic stress generally up-regulates hypothalamic *crh* and pituitary *pomc* expression, along with circulating ACTH levels. Both predictable (25) and unpredictable chronic stress (4, 20) upregulated *crh* mRNA abundance in the pre-optic area. Predictable stress elevated ACTH levels (25), while unpredictable stress variably affected *pomca1* and *pomcb* expression in the pituitary, with reports of upregulation (4) or no effect (20). In Madaro et al. (4), increased *crh* mRNA abundance coincided with *pomca1* and *pomcb* upregulation but did not raise cortisol levels. Instead, chronically stressed fish exhibited a blunted cortisol response to an additional stressor, along with reduced *crfr1*, *pomca1*, and *pomcb* mRNA abundance in the pituitary. The impact of stress duration and protocol type is further supported by zebrafish studies, where different protocols yielded varying cortisol and brain expression profiles (26, 51, 52).

The head kidney, as the primary site of cortisol production in fish, is subject to feedback control, which in the case of mammals has been shown to be regulated by the corticosteroid receptors (53). As acute stress induces an intense cortisol surge from the interrenal tissue, it has been shown in E. sea bass that the tissue’s capacity to release more cortisol is reduced (54). Therefore, the role of the receptors may be crucial to the regulation of a feedback mechanism. In the current study, a down-regulation was observed in the expression of all cortisol receptors after acute stress, regardless of the exposure to chronic stress. The observed down-regulation of *gr1*, *gr2*, and *mr* in the head kidney may reflect a local desensitization mechanism to cortisol, potentially modulating feedback sensitivity within the HPI axis. Literature in fish is limited, but previous studies have shown either decrease in the expression of *mr* in rainbow trout exposed to confinement stress (16), no change in Atlantic salmon (4) or increase in *gr* in gilthead seabream after hypoxia stress (55). The even lower mRNA abundance of *gr2* in chronically stressed fish may be the result of a protective mechanism to prevent overstimulation and maintain homeostasis. Additionally, the unchanged *hsd11b2* mRNA abundance suggests that cortisol inactivation to cortisone remains intact, implying that feedback dysregulation alone does not fully explain the attenuated stress response. Finally, the reduced *pomc* mRNA abundance in the brain of chronically stress fish was not accompanied by changes in *mc2r* mRNA abundance, which encodes the ACTH receptor, at the head kidney. This suggests a lack of compensatory regulation at the adrenal level, consistent with the blunted cortisol response in chronically stressed fish. These findings highlight how chronic stress alters neuroendocrine signaling, potentially compromising the ability to cope with subsequent stressors.

Analysis of the glucocorticoid receptors in the liver pointed out that chronic stress affected their mRNA abundance, being higher for *gr2* and the ratio *gr2/mr* in pre-acute stress conditions. The expression of *gr1* was down-regulated after acute stress in chronically stressed fish only. The above indicate an upregulation of the glucocorticoid receptor in the liver as a result of chronic stress, that could be associated with a need of optimized regulation of the metabolic processes enhancing energy use required to counteract the repeated exposure to stressors (22, 35). Moreover, the mRNA abundance of *hsd11b2* was higher in the liver of chronically stressed fish compared to non-chronically stressed fish, before the application of an additional stressor. The enzyme encoded by this gene regulates the conversion of cortisol to inactive cortisone. However, overall, the levels of circulatory cortisone in chronically stressed fish were lower than those without exposure to chronic stress. These findings suggest that the upregulation of *hsd11b2* mRNA abundance in the former group may serve as a mechanism to limit cortisol activity specifically in the liver during chronic stress. This could also explain the increased mRNA abundance of glucocorticoid receptors, as reduced cortisol availability due to HSD11B2 activity may drive their upregulation. Similarly, following acute stress, the mRNA abundance of *hsd11b2* decreases and this can hypothetically lead to less cortisol inactivation, and therefore higher cortisol levels. Consequently, locally elevated cortisol levels may downregulate glucocorticoid receptor mRNA abundance.

At the gills, chronic stress alone did not affect cortisol receptor gene expression. However, acute stress significantly down-regulated *gr2* mRNA abundance and reduced the *gr2/mr* ratio in both chronic stress groups. Additionally, there was an interaction effect between chronic and acute stress on *gr1* and *mr* mRNA abundance, with blunted expression in acutely stressed fish that had previously experienced chronic stress compared to those without prior exposure. The reduced *gr1* and *mr* mRNA abundance in acutely stressed fish that had undergone chronic stress may reflect a compensatory mechanism in response to repeated activation of the stress axis. This could limit excessive cortisol signaling, preventing potential negative effects of prolonged glucocorticoid exposure, such as impaired osmoregulation (56, 57). In fact, in the complementary study by Samaras et al. (18), plasma osmolality was similar between chronically stress and control fish, either prior or after the exposure to acute stress.

Together the results presented in this study highlight the regulation at the mRNA abundance level of HPI-axis-related and peripheral tissues like liver and gills after exposure to chronic stress in E. sea bass. The authors recognize that the current results should serve a starting point to further dig into the effects of chronic stress in this species. Specifically, one limitation of the study is that mRNA analysis was conducted on whole-brain samples rather than on specific brain regions involved in stress regulation. This may have obscured localized changes in gene expression relevant to the stress response; however, the whole-brain approach still provides a valuable overview of global transcriptional trends and lays the groundwork for more region-specific investigations in future studies. Moreover, while this study provides molecular insights into stress regulation through mRNA abundance analysis, it does not directly measure the protein levels or the receptor functionality. This is especially relevant to interpreting the *gr/mr* ratios, which represent transcript abundance rather than the functional state of the receptors. In addition, in terms of post-acute stress dynamics, the study focuses on mRNA abundance and hormonal responses at specific time points. However, stress responses are dynamic, and additional time-series analyses could help determine whether the observed changes persist or recover over time. Last, but not least, as clearly mentioned, this study focused on the effects of predictable chronic stress and that the nature of the chronic stressor can significantly affect the animal’s responses. In this context, future studies should focus on comparing predictable and unpredictable stress effects, since this could enhance understanding of stress coping mechanisms.

## 5 Conclusions

This study provides novel insights into the physiological and molecular adaptations of E. sea bass to predictable chronic stress, particularly regarding the regulation of cortisol, cortisone, and HPI-axis gene expression across multiple tissues. Although chronic stress did not increase basal cortisol levels, it blunted the acute stress-induced cortisol surge and altered corticosteroid receptor expression. The brain response was affected by both chronic and acute stress, clustering separately the acutely stressed control fish compared to the other groups. Also, the reduced mRNA abundance of *pomc* in the brain of chronically stressed fish and blunted *bdnf* and *gr2/mr* mRNA abundance in chronically stressed fish exposed to an additional acute stressor highlights a habituation of this tissue to repeated exposure to stress. In addition, the reduced *gr2* mRNA abundance and *gr2/mr* ratio in the head kidney and gills suggest a potential mechanism of HPI-axis desensitization, limiting excessive cortisol signaling. Additionally, the increased hepatic mRNA abundance of *gr2* and *hsd11b2* before acute stress highlights a possible tissue-specific metabolic adaptation to chronic stress. The observed modifications in receptor mRNA abundance, particularly in response to acute stress, emphasize the complexity of stress regulation and suggest that chronic stress may prime fish for altered endocrine responses to subsequent challenges. These findings contribute to a broader understanding of how chronic stress influences neuroendocrine function in fish, with potential implications for aquaculture, fisheries management, and stress physiology research. Future studies should explore the long-term effects of these molecular changes on fish performance, resilience, and overall health.

## Data Availability

The raw data supporting the conclusions of this article will be made available by the authors, without undue reservation.

## References

[1] Minuer YS Belzung C Crusio WE. Effects of unpredictable chronic mild stress on anxiety and depression-like behavior in mice. Behav Brain Res. (2006) 175:43–50. doi: 10.1016/j.bbr.2006.07.029 17023061

[2] McEwen BS. Neurobiological and systemic effects of chronic stress. Chronic Stress. (2017) 1. doi: 10.1177/2470547017692328 PMC5573220 28856337

[3] Schreck CB Tort L. The concept of stress in fish. In: Schreck CB Tort L Farrell AP Brauner J, editors. Fish Physiology Vol. 35, Biology of Stress in Fish. Academic Press, Cambridge (2016). p. 1–34.

[4] Madaro A Olsen RE Kristiansen TS Ebbesson LOE Nilsen TO Flik G. Stress in Atlantic salmon: response to unpredictable chronic stress. J Exp Biol. (2015) 218:2538–50. doi: 10.1242/jeb.120535 26056242

[5] Samaras A Santo-Espirito C Papandroulakis N Mitrizakis N Pavlidis M Höglund E. Allostatic load and stress physiology in European seabass (*Dicentrarchus labrax* L.) and gilthead seabream (*Sparus aurata* L.). Front Endocrinol. (2018) 9:451. doi: 10.3389/fendo.2018.00451 PMC6104477 30158900

[6] Tort L. Stress and immune modulation in fish. Dev Comp Immunol. (2011) 35:1366–75. doi: 10.1016/j.dci.2011.07.002 21782845

[7] Cerqueira M Millot S Felix A Silva T Oliveira GA Oliveira CCV. Cognitive appraisal in fish: stressor predictability modulates the physiological and neurobehavioural stress response in sea bass. Proc R Soc. (2020) 287. doi: 10.1098/rspb.2019.2922 PMC7126027 32183629

[8] Barton C. Stress in fishes: a diversity of responses with particular reference to changes in circulating corticosteroids. Integr Comp Biol. (2002) 42:517–25. doi: 10.1093/icb/42.3.517 21708747

[9] Gorissen M Flik G. The endocrinology of the stress response in fish—An adaptation-physiological view. In: Schreck CB Tort L Farrell AP Brauner J, editors. Fish Physiology Vol. 35, Biology of Stress in Fish. Academic Press, Cambridge (2016). p. 75–111.

[10] Mommsen TP Vijayan MM Moon TW. Cortisol in teleosts: dynamics, mechanisms of action, and metabolic regulation. Rev Fish Biol Fish. (1999) 9:211–68. doi: 10.1023/A:1008924418720

[11] Fanouraki E Mylonas CC Papandroulakis N Pavlidis M. Species specificity in the magnitude and duration of the acute stress response in Mediterranean marine fish in culture. Gen Comp Endocrinol. (2011) 173:313–22. doi: 10.1016/j.ygcen.2011.06.004 21712040

[12] Guo H Dixon B. Understanding acute stress-mediated immunity in teleost fish. Fish Shellfish Immunol Rep. (2021) 2:100010. doi: 10.1016/j.fsirep.2021.100010 36420509 PMC9680050

[13] Aerts J Metz JR Ampe B Decostere A Flik G De Saeger S. Scales tell a story on the stress history of fish. PloS One. (2015) 10:e0123411. doi: 10.1371/journal.pone.0123411 25922947 PMC4414496

[14] Carbajal A Reyes-Lopez FE Tallo-Parra O Lopez-Bejar M Tort L. Comparative assessment of cortisol in plasma, skin mucus and scales as a measure of the hypothalamic-pituitary-interrenal axis activity in fish. Aquaculture. (2019) 506:410–16. doi: 10.1016/j.aquaculture.2019.04.005

[15] Kennedy EKC Janz DM. Chronic stress causes cortisol, cortisone and DHEA elevations in scales but not serum in rainbow trout. Comp Biochem Physiol Part A. (2023) 276:111352. doi: 10.1016/j.cbpa.2022.111352 36427661

[16] Kiilerich P Servili A Péron S Valotaire C Goardon L Leguen I. Regulation of the corticosteroid signalling system in rainbow trout HPI axis during confinement stress. Gen Comp Endocrinol. (2018) 258:184–93. doi: 10.1016/j.ygcen.2017.08.013 28837788

[17] Magierecka A Lind AL Aristeidou A Sloman KA Metcalfe NB. Chronic exposure to stressors has a persistent effect on feeding behaviour but not cortisol levels in sticklebacks. Anim Behav. (2021) 181:71–81. doi: 10.1016/j.anbehav.2021.08.028

[18] Samaras A Dimitroglou A Kollias S Skouradakis G Papadakis IE Pavlidis M. Cortisol concentration in scales is a valid indicator for the assessment of chronic stress in European sea bass, *Dicentrarchus labrax* L. Aquaculture. (2021) 545:737257. doi: 10.1016/j.aquaculture.2021.737257

[19] Houdelet C Blondeau−Bidet E Mialhe X Lallement S Devilliers. S Falguière JC. Plasma cortisol and production of miRNAs in red drum (*Sciaenops ocellatus*) exposed to three distinct challenges. Fish Physiol Biochem. (2024) 50:757–66. doi: 10.1007/s10695-024-01304-x 38265685

[20] Opinion AGR Vanhomwegen M De Boeck G Aerts J. Long-term stress induced cortisol downregulation, growth reduction and cardiac remodeling in Atlantic salmon. J Exp Biol. (2023) 226:jeb246504. doi: 10.1242/jeb.246504 37921456 PMC10690108

[21] Xu C Su L Qiu N Hou M Yu F Zou X. The effect of unpredictable chronic stress on rare minnow (*Gobiocypris rarus*): growth, behaviour and physiology. Biology. (2022) 11:1755. doi: 10.3390/biology11121755 36552265 PMC9775413

[22] Lopes T Costas B Ramos-Pinto L Reynolds P Imsland AKD Aragão C. Lumpfish physiological response to chronic stress. Front Mar Sci. (2024) 11:1443710. doi: 10.3389/fmars.2024.1443710

[23] Patel DM Brinchmann MF Hanssen A Iversen MH. Changes in the skin proteome and signs of allostatic overload type 2, chronic stress, in response to repeated overcrowding of lumpfish (*Cyclopterus lumpus* L.). Front Mar Sci. (2022) 9:891451. doi: 10.3389/fmars.2022.891451

[24] Quadros VA Rosa LV Costa FV Koakoski G Barcellos LJG Rosemberg DB. Predictable chronic stress modulates behavioral and neuroendocrine phenotypes of zebrafish: Influence of two homotypic stressors on stress-mediated responses. Comp Biochem Physiol Part C. (2021) 247:109030. doi: 10.1016/j.cbpc.2021.109030 33722767

[25] Virtanen MI Brinchmann MF Patel DM Iversen MH. Chronic stress negatively impacts wound healing, welfare, and stress regulation in internally tagged Atlantic salmon (*Salmo salar*). Front Physiol. (2023) 14:1147235. doi: 10.3389/fphys.2023.1147235 37078022 PMC10106625

[26] Pavlidis M Theodoridi A Tsalafouta A. Neuroendocrine regulation of the stress response in adult zebrafish, *Danio rerio* . Prog Neuro Psychopharmacol Biol Psychiatry. (2015) 60:121–31. doi: 10.1016/j.pnpbp.2015.02.014 25748166

[27] Virtanen MI Iversen MH Patel DM Brinchmann MF. Daily crowding stress has limited, yet detectable effects on skin and head kidney gene expression in surgically tagged atlantic salmon (*Salmo salar*). Fish Shellfish Immunol. (2024) 152:109794. doi: 10.1016/j.fsi.2024.109794 39089638

[28] Best C Gilmour KM. Regulation of cortisol production during chronic social stress in rainbow trout. Gen Comp Endocrinol. (2022) 325:114056. doi: 10.1016/j.ygcen.2022.114056 35594954

[29] Numakawa T Odaka H Adachi N. Actions of brain-derived neurotrophic factor and glucocorticoid stress in neurogenesis. Int J Mol Sci. (2017) 18:2312. doi: 10.3390/ijms18112312 29099059 PMC5713281

[30] Tsimpolis A Kalafatakis K Charalampopoulos I. Recent advances in the crosstalk between the brain-derived neurotrophic factor and glucocorticoids. Front Endocrinol. (2024) 5:1362573. doi: 10.3389/fendo.2024.1362573 PMC11027069 38645426

[31] Vindas MA Fokos S Pavlidis M Hoglund E Dionysopoulou S Ebbesson LOE. Early life stress induces long-term changes in limbic areas of a teleost fish: the role of catecholamine systems in stress coping. Sci Rep. (2018) 8:5638. doi: 10.1038/s41598-018-23950-x 29618742 PMC5884775

[32] Kirsten K Pompermaier A Koakoski G Mendonca-Soares S Angnes da Costa R Maffi VC. Acute and chronic stress differently alter the expression of cytokine and neuronal markers genes in zebrafish brain. Stress. (2020) 24:107–12. doi: 10.1080/10253890.2020.1724947 32013653

[33] Nematollahi MA van Pelt-Heerschap H Atsma W Komen J. High levels of corticosterone, and gene expression of star, cyp17a2, hsd3b, cyp21, hsd11b2 during acute stress in common carp with interrenal hyperplasia. Gen Comp Endocrinol. (2012) 176:252–8. doi: 10.1016/j.ygcen.2012.01.023 22333211

[34] Samaras A Pavlidis M. Regulation of divergent cortisol responsiveness in European sea bass, *Dicentrarchus labrax* L. PloS One. (2018) 13:e0202195. doi: 10.1371/journal.pone.0202195 30096195 PMC6086447

[35] Hernández-Pérez J Naderi F Chivite M Soengas JL Míguez JM López-Patiño MA. Influence of stress on liver circadian physiology. A study in rainbow trout, *Oncorhynchus mykiss*, as fish model. Front Physiol. (2019) 10:611. doi: 10.3389/fphys.2019.00611 31164837 PMC6536609

[36] de Kloet ER Oitzl MS Joels M. Stress and cognition: are corticosteroids good or bad guys? Trends Neurosci. (1999) 22:422–26. doi: 10.1016/s0166-2236(99)01438-1 10481183

[37] Johansen IB Sandvik GK Nilsson GE Bakken M Øverli Ø. Cortisol receptor expression differs in the brains of rainbow trout selected for divergent cortisol responses. Comp Biochem Physiol D. (2011) 6:126–32. doi: 10.1016/j.cbd.2010.11.002 21220219

[38] Samaras A Pavlidis M Lika K Theodoridi A Papandroulakis N. Scale matters: performance of European sea bass, *Dicentrarchus labrax*, L., (1758) reared in cages of different volumes. Aquacult Res. (2017) 48:990–1005. doi: 10.1111/are.12942

[39] Tsalafouta A Papandroulakis N Gorissen M Katharios P Flik G Pavlidis M. Ontogenesis of the HPI axis and molecular regulation of the cortisol stress response during early development in *Dicentrarchus labrax* . Sci Rep. (2014) 4:5525. doi: 10.1038/srep05525 24984570 PMC4078316

[40] Pavlidis M Karantzali E Fanouraki E Barsakis C Kollias S Papandroulakis N. Onset of the primary stress in European sea bass *Dicentrarhus labrax*, as indicated by whole body cortisol in relation to glucocorticoid receptor during early development. Aquaculture. (2011) 315:125–30. doi: 10.1016/j.aquaculture.2010.09.013

[41] Tsalafouta A Gorissen M Pelgrim TNM Papandroulakis N Flik G Pavlidis M. α-MSH and melanocortin receptors at early ontogeny in European sea bass (*Dicentrarchus labrax*, L.). Sci Rep. (2017) 7:46075. doi: 10.1038/srep46075 28378841 PMC5380957

[42] Vandesompele J de Preter K Pattyn F Poppe B van Roy N de Paepe A. Accurate normalization of real-time quantitative RT-PCR data by geometric averaging of multiple internal control genes. Genome Biol. (2002) 3:research0034.1. doi: 10.1186/gb-2002-3-7-research0034 12184808 PMC126239

[43] Patino R Redding MJ Schreck CB. Interrenal secretion of corticosteroids and plasma cortisol and cortisone concentrations after acute stress and during seawater acclimation in juvenile coho salmon (*Oncorhynchus kisutch*). Gen Comp Endocrinol. (1987) 68:431–9. doi: 10.1016/0016-6480(87)90082-7 3436517

[44] Pottinger TG Moran TA. Differences in plasma cortisol and cortisone dynamics during stress in two strains of rainbow trout (*Oncorhynchus mykiss*). J Fish Biol. (1993) 43:121–30. doi: 10.1111/j.1095-8649.1993.tb00415.x

[45] Sturm A Bury NR. Duplicate teleost glucocorticoid receptors are not functionally redundant: relationship between structural and functional differences. Comp Biochem Physiol A. (2005) 141:S208.

[46] Prunet P Sturm A Milla S. Multiple corticosteroid receptors in fish: from old ideas to new concepts. Gen Comp Endocrinol. (2006) 147:17–23. doi: 10.1016/j.ygcen.2006.01.015 16545810

[47] Stolte EH de Mazon AF Leon-Koosterziel KM Jęsiak M Bury NR Sturm A. Corticosteroid receptors involved in stress regulation in common carp, *Cyprinus carpio* . J Endocrinol. (2008) 198:403–17. doi: 10.1677/JOE-08-0100 18505847

[48] Joëls M Karst H DeRijk R De Kloet ER. The coming-out of the brain mineralocorticoid receptor. Trends Neurosci. (2008) 31:1–7. doi: 10.1016/j.tins.2007.10.005 18063498

[49] Korte SM Olivier B Koolhaas JM. A new animal welfare concept based on allostasis. Physiol Behav. (2007) 92:422–8. doi: 10.1016/j.physbeh.2006.10.018 17174361

[50] Cerqueira M Millot S Silva T Felix A Castanheira MF Rey S. Stressor controllability modulates the stress response in fish. BMC Neurosci. (2021) 22:48. doi: 10.1186/s12868-021-00653-0 34348667 PMC8336412

[51] Demin KA Taranov AS Ilyin NP Lakstygal AM Volginc AD de Abreu MS. Understanding neurobehavioral effects of acute and chronic stress in zebrafish. Stress. (2021) 24:1–18. doi: 10.1080/10253890.2020.1724948 32036720

[52] Piato AL Capiotti KM Tamborski AR Oses JP Barcellos LJG Bogo MR. Unpredictable chronic stress model in zebrafish (*Danio rerio*): Behavioral and physiological responses. Prog Neuro Psychopharmacol Biol Psychiatry. (2011) 35:561–7. doi: 10.1016/j.pnpbp.2010.12.018 21187119

[53] Gjerstad JK Lightman SL Spiga F. Role of glucocorticoid negative feedback in the regulation of HPA axis pulsatility. Stress. (2018) 21(5):403–16. doi: 10.1080/10253890.2018.1470238 PMC6220752 29764284

[54] Rotllant J Ruane NM Caballero MJ Montero D Tort L. Response to confinement in sea bass (Dicentrarchus labrax) is characterised by an increased biosynthetic capacity of interrenal tissue with no effect on ACTH sensitivity. Comp. Biochem. Physiol. A Mol. Integr. Physiol. (2003) 136:613–20. doi: 10.1016/s1095-6433(03)00211-3 14613789

[55] Garcia-Meilan I Tort L Khansari AR. Rainbow trout integrated response after recovery from short-term acute hypoxia. Front Physiol. (2022) 13:1021927. doi: 10.3389/fphys.2022.1021927 36338491 PMC9634174

[56] Laiz-Carrion R Martin del Rio MP Miguez JM Mancera JM Soengas JL. Influence of cortisol on osmoregulation and energy metabolism in gilthead seabream *Sparus aurata* . J Exp Zool. (2003) 298A:105–18. doi: 10.1002/jez.a.10256 12884272

[57] Vargas-Chacoff L Regish AM Weinstock A Bjornsson BT McCormick SD. Effects of long-term cortisol treatment on growth and osmoregulation of Atlantic salmon and brook trout. Gen Comp Endocrinol. (2021) 308:113769. doi: 10.1016/j.ygcen.2021.113769 33794274

